# Surface-Enhanced IR-Absorption Microscopy of *Staphylococcus aureus* Bacteria on Bactericidal Nanostructured Si Surfaces

**DOI:** 10.3390/molecules24244488

**Published:** 2019-12-07

**Authors:** Sergey I. Kudryashov, Alena A. Nastulyavichus, Eteri R. Tolordava, Alexey N. Kirichenko, Irina N. Saraeva, Andrey A. Rudenko, Yulia M. Romanova, Andrey Yu. Panarin, Andrey A. Ionin, Tatiana E. Itina

**Affiliations:** 1 Lebedev Physical Institute, 119991 Moscow, Russia; ganuary_moon@mail.ru (A.A.N.); tolordava.eteri@yandex.ru (E.R.T.); heddwch@mail.ru (I.N.S.); aa_rudenko@mail.ru (A.A.R.); ioninaa@lebedev.ru (A.A.I.); 2 ITMO University, 197101 St. Petersburg, Russia; 3 N.F. Gamaleya Federal Research Centre of Epidemiology and Microbiology, 123098 Moscow, Russia; genes2007@yandex.ru; 4 FSBI TISNCM, 108840 Moscow, Russia; akir73@mail.ru; 5 B.I. Stepanov Institute of Physics NAS Belarus, 220072 Minsk, Belarus; a.panarin@ifanbel.bas-net.by; 6 Laboratoire Hubert Curien, UMR CNRS 5516/UJM/Univ. Lyon, 42000 Saint-Etienne, France; tatiana.itina@univ-st-etienne.fr

**Keywords:** Si nanostructures, staphylococcus aureus bacterial biofilm, bactericidal effect, surface-enhanced IR microscopy, chemical enhancement

## Abstract

Surface-enhanced IR absorption (SEIRA) microscopy was used to reveal main chemical and physical interactions between *Staphylococcus aureus* bacteria and different laser-nanostructured bactericidal Si surfaces via simultaneous chemical enhancement of the corresponding IR-absorption in the intact functional chemical groups. A cleaner, less passivated surface of Si nanoripples, laser-patterned in water, exhibits much stronger enhancement of SEIRA signals compared to the bare Si wafer, the surface coating of oxidized Si nanoparticles and oxidized/carbonized Si (nano) ripples, laser-patterned in air and water. Additional very strong bands emerge in the SEIRA spectra on the clean Si nanoripples, indicating the potential chemical modifications in the bacterial membrane and nucleic acids during the bactericidal effect.

## 1. Introduction

Nanostructured surfaces of different materials exhibit unexpected and unprecedented bactericidal effects regarding pathogenic bacteria, which is usually related either to photochemical reaction of gaseous oxygen with composing nanocrystallites, yielding in reactive oxygen species and singlet oxygen [1,2,3,4,5], or to mechanical reaction of bacterial membranes to sharp surface nanoroughness [6,7,8]. Specifically, in the case of Si a broad variety of its nanomorph–nanospike [6], nanosheets [7] and nanoparticles (NPs) [9] were demonstrated to be bactericidal.

Moreover, surface-enhanced Raman scattering (SERS) and IR-absorption (reflection) (SEIRA/R) was performed on nanostructured Si surfaces, where linear- and non-linear optical enhancement could be achieved via plasmonic enhancement of local electric fields in heavily doped Si [10], magnetic dipolar resonance [11] or chemical charge-transfer processes. This increased the dipolar polarity between interacting surface and molecule functional groups [12], despite the fact that such a nanostructured material is sometimes considered as chemically inert [13]. Highly sensitive visible and near-IR SERS probes surface-adsorbed molecules only within the nanoscale interfacial region of the optical-range electrical near fields, making available for detection only the exterior elements of (sub) micro-scale viruses and bacteria [14,15,16]. In contrast, less-sensitive mid- and far-IR (typical wavelengths ~3–25 microns) SEIRA(R) probing involves the micro-scale interfacial region of the IR electrical near fields, being highly beneficial for characterization of specific internal elements in viruses and bacteria [17,18]. As a result, it is SEIRA(R) that potentially holds promise as a very important spectroscopic modality in characterizing key-enabling interactions of pathogenic bacteria and bactericidal nanostructured surfaces.

The main objective of this particular study was to use IR-microscopy of *Staphylococcus aureus* bacterial biofilms on different nanostructured silicon surfaces to reveal key interfacial chemical and physical interactions, and their underlying bactericidal effects. This study is facilitated by chemical enhancement of SEIRA signals via the same bactericidal and abiotic chemical bonding at these interfaces, opening the way for direct cellular and molecular scale analysis of their corresponding bactericidal performance.

## 2. Experimental Results

### 2.1. IR Characterization of Bactericidal Si Nanoripples and Nanoparticle Coatings

**Figure 1:**
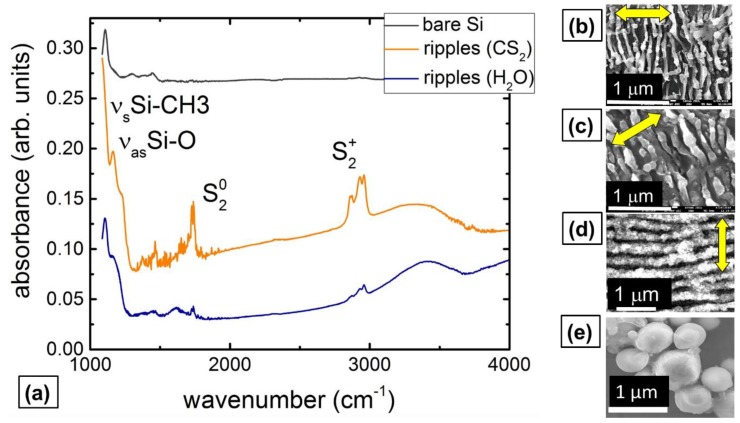
(**a**) IR absorbance spectrum of the bare Si wafer and IR absorbance spectra of nanostructured Si surfaces, normalized to the reference Si spectrum, with their principal band assignment. Insets: top-view SEM images of Si nanostructures laser patterned in CS_2_ (**b**), H_2_O (**c**) and air (**d**), as a well as of Si NP coating (**e**).

The nanopatterned Si spots indicated that weak homogeneous ablation of the wet Si wafers across the focused Gaussian beam was enhanced by strong nanoscale surface plasmon-mediated ablation, providing the polarization-dependent directionality of the generated 1D-nanoripples (Figure 1b–d). In both these fluids (H_2_O, CS_2_), the nanoripples appear as homogeneous regular arrays of ultra-thin Si 2D-nanosheets (thickness down to 30 nm) with their “normal” orientation perpendicular to the laser polarization, similarly to common surface ripples [19,20] but with the extraordinarily small period Λ ≈ 0.1 μm ~ λ/10 and the extraordinarily tall (<1 μm, aspect ratio ~ 5) [7]. For comparison, the corresponding minimal ripple period in air approaches to Λ(air) ≈ 0.9 μm at the same normal orientation regarding the laser polarization (Figure 1d). Moreover, these nanoripples are tunable in their period, exhibiting considerable (30%–40%) reduction of their periods versus the cumulative laser exposure N—from Λ(H_2_O) ≈ 0.18 μm and Λ(CS_2_) ≈ 0.16 μm at N = 100 until Λ(H_2_O) ≈ 0.12 μm and Λ(CS_2_) ≈ 0.1 μm at N = 2400.

**Figure 2:**
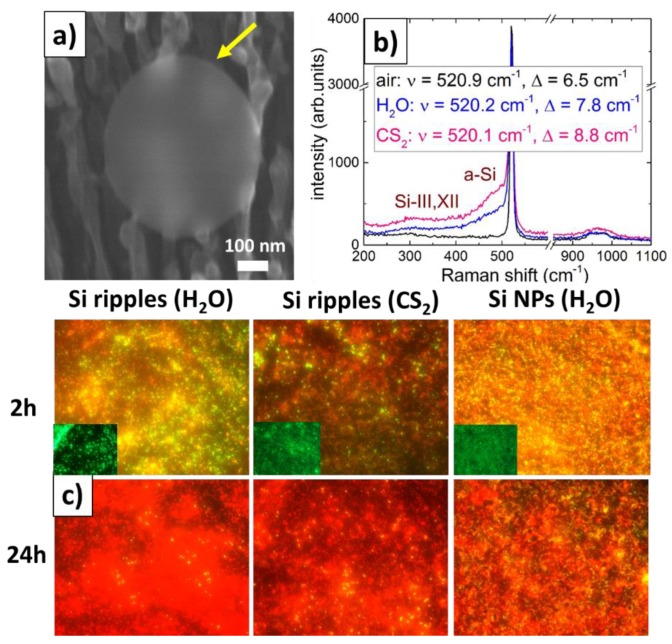
(**a**) Top-view SEM image of the nanosharp Si ripple pattern with the single inactivated *Staphylococcus aureus* bacterium (the bead marked by the yellow arrow). (**b**) Raman spectra of diverse Si surface ripples at the 488 nm excitation with their spectral and structural parameters (see the text above). (**c**) Images of stained live “green” (insets) and dead “red” *Staphylococcus aureus* bacteria on the Si nanopatterns and nanocoatings after 2 h and 24 h incubation. The frame sizes are 60 × 90 μm.

Fast Fourier-transform (FFT) IR micro-spectroscopy of the nanopatterned spots indicates considerable oxidation of the Si surfaces in Figure 1a—weak for the bare Si wafer, as shown by its absorbance spectrum with oxygen asymmetric stretching vibrational bands at 1075 and 1150 cm^−1^ [21]. In contrast, surprisingly the Si surface nanopatterns produced in carbon disulfide demonstrate much stronger oxygen asymmetric stretching vibrational bands at 1075 and 1150 cm^−1^. Apparently, this is considerably contributed to by Si-CH_2_-Si wagging and CH_3_ symmetrical deformation in Si-CH_3_ at 1020–1090 and 1250–1260 cm^−1^ [22], respectively. The additional strong bands at 1730 (0.21 eV) and 2860–2960 (0.37 eV) cm^−1^ correspond to, respectively, the dimer states of donor sulfur impurity in neutral (S_2_^0^) and charged (S_2_^+^) states, lying at 0.188 and 0.37 eV [23]. Meanwhile, elemental compositions of the patterns acquired by energy-dispersion X-ray spectroscopy (EDX) at 10 keV (Table 1), strongly support our IR band assignment and analysis above, demonstrating for carbon disulfide ambient (CS_2_) very significant relative contents of oxygen, carbon and sulfur–10 (O), 14 (C) and 1 (S) at. %, comparing to the water ambient (H_2_O)–2 (O) and 6 (C) at. %, possibly, because of a film boiling isolation of the Si surface. Moreover, fs-laser patterning of Si in ambient air at the same laser and scanning parameters exhibits even stronger chemical modification–15 (O) and 17 (C) at. % (not shown), similarly to Si NPs produced by nanosecond laser ablation of the Si wafer in water (Figure 1e). All three types of patterns demonstrate a loss of structural order—LO/TO-line shift and broadening, presence of amorphous (a-Si) and high-pressure phases Si-III,XII [24] in Figure 2b, which is more pronounced for the wet patterning, specifically in CS_2_.

### 2.2. IR Characterization of Staphylococcus Aureus Bacterial Biofilms on Bactericidal Si Nanoripples and Nanoparticle Coatings

Recently, a fascinating “nanomechanical” bactericidal effect of Si surface nanoroughness was demonstrated relative to diverse pathogens [6,8] being, potentially, accompanied or enhanced by chemical effect of singlet oxygen and other reactive oxygen species generated on illuminated Si surfaces [1,3]. In this study, despite the initial modest bactericidal response (red coloration due to the cell membrane damage upon 2 h incubation, Figure 2c) for all these samples, the long-term (24 h) effect appears to be more pronounced for the cleanest nanoripples produced in H_2_O, while less pronounced for the disordered and chemically-modified nanoripples produced in liquid CS_2_, comparing to the even more minor effect for Si NPs. The observed bactericidal effect could occur owing to their good chemical binding to the clean nanostructured Si surface, unprecedented sharpness of the Si nanoripples, and, potentially, amorphous Si and sulfur contents, surface oxidation and carbonization, thus requiring the following research and analysis.

**Figure 3:**
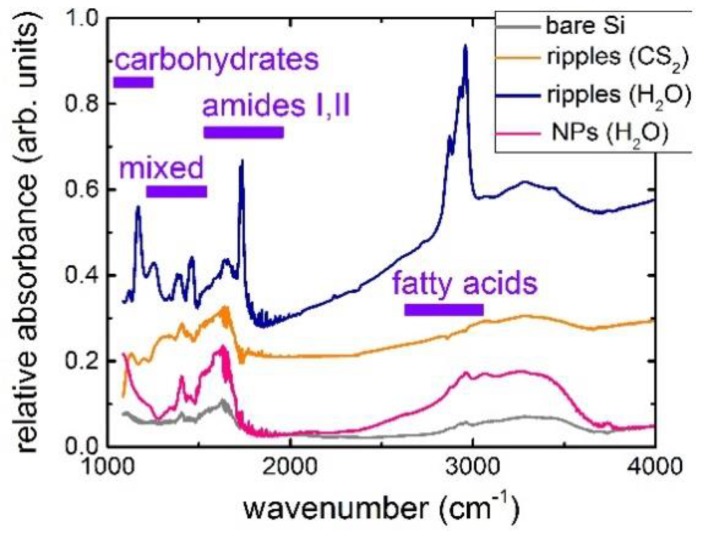
IR spectra of relative absorbance of the *Staphylococcus aureus* bacterial films on the nanostructured Si surfaces, normalized to their corresponding transmittances without bacterial films, with their principal band assignment after [22] (control—*Staphylococcus aureus* bacterial film on the bare smooth Si wafer). The upper spectrum is offset up by 0.15 for clarity.

FFT-IR micro-spectroscopy of the *Staphylococcus aureus* bacterial biofilms on the nanostructured Si surfaces was performed with the control on the abiotic bare Si wafer surface (Figure 3). In this case, the relative absorbance of the bacterial films exhibits a number of typical bands [25]:

- in the window between 3000 and 2800 cm^−1^ (W_1_, the ‘fatty acid region I’), dominated by the -CH_3_, >CH, and =CH stretching vibrations of the functional groups usually present in the fatty acid components of the various membrane amphiphiles;
- in the window between 1800 and 1500 cm^−1^ (W_2_, the ‘amide region’), dominated by the amide I and amide II bands of proteins and peptides;
- in the window between 1500 and 1200 cm^−1^ (W_3_, the ‘mixed region’), a spectral region containing information from proteins, fatty acids and phosphate-carrying compounds, including the window between 1500 and 1400 cm^−1^ (W_31_, the ‘fatty acid region II’), dominated by the -CH_3_ and -CH_2_ bending vibrations of the same functional groups as expressed in W_1_;
- in the window between 1200 and 900 cm^−1^ (W_4_, the ‘polysaccharide region’), dominated by the fingerprint-like absorption bands of the carbohydrates present within the cell wall.

On the bare Si substrate, the bacterial film demonstrates a rather weak own relative IR absorbance of all the characteristic bands, accounting for the corresponding IR transmittance of the underlying Si substrate.

**Figure 4:**
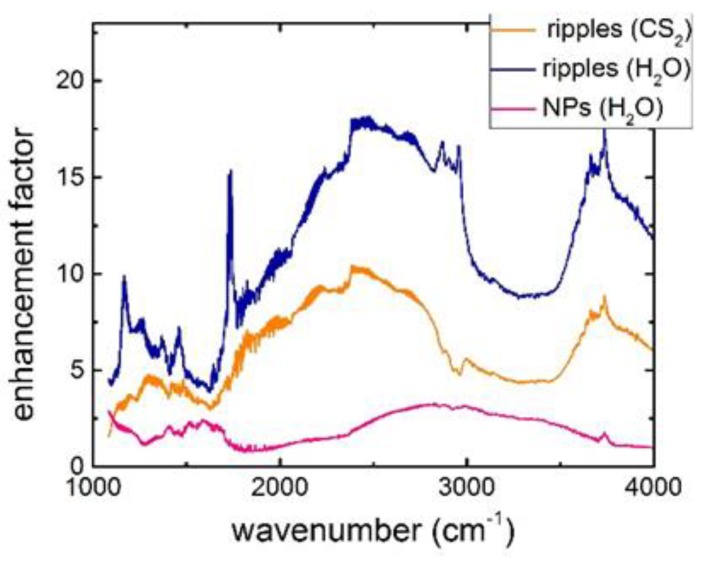
Enhancement factors for IR absorption in the *Staphylococcus aureus* bacterial films on the different nanostructured Si surfaces, normalized to the control absorbance spectrum of the same bacterial film on the smooth bare Si wafer.

In contrast, all these Si surface nanopatterns exhibit much stronger IR absorbance for all the characteristic absorption bands of *Staphylococcus aureus* bacteria (Figure 3), which is not the influence of their hydrophilic surface wetting (contact angles in the range of ~40–70°). It appears 2–3 fold more intense for Si-NP coating (Figure 4), 5–10 fold more intense for the Si nanopatterns produced in carbon disulfide, and 5–15 fold more intense for the less oxidized and carbonized Si nanopatterns produced in water. Since these characteristic absorption bands of *Staphylococcus aureus* bacteria are related above to the chemically active functional groups (fatty acids, amides, polycarbonates, proteins, peptides) of the Gram-positive cell membrane, their enhanced absorbance can be assigned to intense chemical interactions with the nanostructured surfaces. Such additional chemical bonding results in the interfacial chemical enhancement of the IR absorbance of *Staphylococcus aureus* bacteria in the SEIRA arrangement (Figure 4), more pronounced for the less passivated (oxidized, carbonized) Si surface nanopatterned in water.

Finally, a few strong and rather narrow, novel, additional absorption bands appeared in the IR absorbance spectra of *Staphylococcus aureus* bacteria at 1170 and 1740 cm^−1^ on the Si surfaces patterned in water (Figure 3), comparing even to previous UV-laser irradiation of such bacterial films [26]. These specific bands exhibit two orders of magnitude stronger absorbance (relative to the corresponding bands in the control experiments without background), emerging in the same characteristic absorption bands of *Staphylococcus aureus* bacterial membrane. However, the new emerging band at 1170 cm^−1^ can be related to δ (COP), C-C and COH-vibrations in deoxyribonucleic acid (DNA) and ribonucleic acid (RNA) backbones [26], while the specific new band at 1740 cm^−1^ can be linked to >C=O vibrations in lipid esters [26] or to peroxide oxidation of nucleic acids during oxidative stress, driven by reactive oxygen compounds [27].

Therefore, even though generally detailed principal component analysis of IR spectra of complex molecules or microorganisms is quite ambiguous, our results potentially indicate those chemical functional groups of the bacterial membrane, which predominantly interact with the rather clean nanostructured Si surface, apparently resulting in the bactericidal membrane damage (‘external damage’), as shown by the red coloration of the bacteria in our live/dead tests due to the dye penetration through their membranes. Moreover, there are also some indications of bacterial RNA/DNA interactions with the nanostructured Si surfaces, potentially implying their internal chemical damage.

## 3. Concluding Remarks

In this study, the advanced surface-enhanced IR absorption (SEIRA) microscopic study of basic chemical and physical interactions between *Staphylococcus aureus* bacteria and state-of-the-art nanostructured bactericidal Si surfaces was performed, harnessing simultaneous chemical enhancement of the corresponding IR-absorption in the intact functional chemical groups. Cleaner, less passivated surface of Si nanoripples, laser-patterned in water, was observed to demonstrate much stronger SEIRA enhancement compared to the bare Si wafer, oxidized Si nanoparticles and oxidized/carbonized Si (nano) ripples, laser-patterned in air and water. Additional very strong bands emerge in the SEIRA spectra on the clean Si nanoripples, indicating the potential chemical modifications—both in the bacterial membrane and RNA/DNA backbones, during the bactericidal effect.

## 4. Materials and Methods

Si nanopatterns were fabricated via single-pass scanning of commercial Si (111) wafers in the form of their 2 cm × 2 cm wide and 0.4 mm thick pieces by means of a laser nano/micromachining workstation. The nanopatterning was performed by 1030 nm, 300 fs pulses of an ytterbium-doped fiber laser Satsuma, delivered at 6 μJ pulse energies (TEM_00_ mode) and repetition rate f = 160 kHz, focused onto the sample surface in a glass beaker into a spot with the 1/e-radius σ_1/e_ ≈ 15 μm (the peak fluence ≈ 1 J/cm^2^) through 5 mm thick carbon disulfide (CS_2_) or double-distilled water. Surface scanning was provided by a galvanoscanner ATEKO^TM^ with 100 lines/mm surface filling and 13 μm inter-spot distance in the lines at scan velocity V = 12 mm/s (the corresponding surface exposure N = 2σ_1/e_f_/_V = 400 shots per spot). Surface topography and chemical composition of the nanopatterned samples were characterized by means of a scanning electron microscope (SEM) JEOL 7001F, equipped by an energy-dispersion x-ray spectroscopy (EDX) module INCA (Oxford Instruments, UK) for chemical micro-analysis at the 10 keV kinetic energies of electrons. Raman micro-spectroscopy of the nanopatterned samples was performed at the 488 nm wavelength (U-1000, Jobin Yvon, France).

The anticipated antibacterial effect of the fabricated Si nanopatterns (nanoripples) was tested on the next day on Gram-negative clinical isolate *Staphylococcus aureus* bacteria as biofilms. The method of obtaining bacterial biofilms with its subsequent coloration using “Live/Dead Biofilm Viability Kit” was described elsewhere [7]. Specifically, 18 h culture of the bacteria grown in a nutrient medium (LB), was diluted with fresh LB in the proportion 1:100. A Petri dish with the broth culture was placed for investigation. For biofilm formation, the samples of the Si wafers fs-laser nanopatterned in air, water (H_2_O) and carbon disulfide (CS_2_) ambients, were incubated for 24 h at 37 °C. A “Live/Dead Biofilm Viability Kit” coloration set was used to differentiate between viable and non-viable bacteria in the biofilms. SYTO^®^9 green fluorescent dye (component A) and propidium iodide (component B) red fluorescent dye formed the complete staining set. The staining method is based on the penetration of the two dyes into the bacterial cells. SYTO^®^9 (3.34 mM in DMSO) dye binds to DNA and stains both live and dead bacteria with damaged and intact cell membranes, while propidium iodide (20 mM in DMSO) dye colors dead bacteria with damaged cell membranes. To visualize the bacterial biofilms, the fluorescent dyes: 3 μL SYTO^®^9 and 3 μL propidium iodide were diluted in 1 mL of distilled water. Then, the nutrient medium was removed from the Petri dish and biofilms on the samples were submerged in a solution from the “Live/Dead Biofilm Viability Kit” for 15 min in darkness. After incubation, the samples were immediately washed three times with water and analyzed, using the fluorescence microscope Nikon H600L with a 40× fluorescence objective lens (instrumental magnification—600×). IR-micro-spectroscopy of the nanopatterned samples with and without bacterial films was performed in a transmission mode in the range of 400–7000 cm^−1^ (Thermo Nicolet Nexus 470 FT-IR spectrometer, USA).

For IR-spectroscopic characterization, clinical isolates from the collection of the genetic engineering laboratory of pathogenic microorganisms were used: *Staphylococcus aureus* 15 and *Pseudomonas aeruginosa* 32. Bacterial cultures were grown in Luria-Bertani (LB) liquid medium using standard dry and liquid culture media manufactured by Difco (New York, NY, USA). Then, 1 mL of overnight broth culture of bacteria was placed in a sterile tube and centrifuged for 5 min at 3000× *g*. Then the cultures were washed three times with sterile NaCl, each time separating the cells by centrifugation. Finally, prior to spectroscopy, the resulting bacterial suspensions in NaCl solution, taken in the quantity of 100 μL, were transferred to various substrates and dried.

## Figures and Tables

**Table 1:** Compositional chemical EDX analysis of the nanostructured Si surfaces.

Structure/Chemical Composition (at. %)	Silicon Si	Oxygen O	Carbon C	Sulfur S
Si ripples (air)	67 ± 1	15 ± 1	17 ± 1	0
Si nanoripples (CS_2_)	75 ± 1	10 ± 1	14 ± 1	0.9 ± 0.3
Si nanoripples (H_2_O)	92 ± 1	2 ± 1	6 ± 1	0
Si nanoparticles (H_2_O)	84 ± 1	16 ± 1	0	0
